# Einführung

**DOI:** 10.1007/978-3-662-61522-5_1

**Published:** 2020-11-30

**Authors:** Ulrike Pröbstl-Haider, Dagmar Lund-Durlacher, Gudrun Obersteiner, Franz Prettenthaler, Andrea Damm, Petra Stolba

**Affiliations:** 1 Institut für Landschaftsentwicklung, Erholungs- und Naturschutzplanung, University of Natural Resources and Life Sciences, Wien, Wien Austria; 2 Tourism and Service Management, MODUL University Vienna, Wien, Wien Austria; 3 Klimaforschung, ZAMG, Wien, Wien Austria; 4 Forschungsgesellschaft / LIFE, JOANNEUM RESEARCH, Graz, Steiermark Austria; 5 Institut für Landschaftsentwicklung, Erholungs- und Naturschutzplanung, University of Natural Resources and Life Sciences, Wien, Österreich; 6 MODUL University Vienna, Wien, Österreich; 7 Wien, Österreich; 8 JOANNEUM RESEARCH, Graz, Österreich

## Abstract

Die österreichische Tourismusbranche kann auf viele Jahrzehnte einer wirtschaftlich erfolgreichen Entwicklung zurückblicken und leistet einen wesentlichen Beitrag zur bundesweiten Gesamtwirtschaftsleistung und -beschäftigung. Ein Großteil des Sommer- und Wintertourismus in Österreich ist mit Aktivitäten in der Natur verbunden und somit höchst sensitiv auf Änderungen des Klimas – vor allem hinsichtlich Niederschlagsmengen und Temperaturen. Der Klimawandel hat somit einen großen Einfluss auf die Nachfrage durch Touristinnen und Touristen sowie auf das Angebot an Attraktionen in Österreich (Köberl et al. 2014).

Allgemein stellt der Klimawandel eine der größten Herausforderungen der Menschheit im 21. Jahrhundert dar. Die Lufttemperatur ist in Österreich seit Ende des 19. Jahrhunderts bereits um knapp 2 °C gestiegen, in etwa doppelt so stark wie im globalen Mittel (siehe Kap. 2). Die Einhaltung des Pariser Klimaabkommens, den globalen Temperaturanstieg deutlich unter 2 °C gegenüber dem vorindustriellen Niveau zu halten, ist zwingend erforderlich, um stärkere negative Auswirkungen noch abwenden zu können (UNFCCC 2015). Angesichts der aktuellen Emissionsbilanz der Österreicherinnen und Österreicher mit 15 t $CO_2$-Äquivalenten[1] pro Kopf (im Jahr 2015, Windsperger et al. 2017) – weit entfernt vom Zielwert von nahe null im Jahr 2050 – erfordert dies weitreichende Änderungen.

Die klimatische Veränderung wirkt sich auf den Tourismus in unterschiedlicher Weise aus. Gössling et al. (2012) identifizieren vier Kernaspekte von Auswirkungen des Klimawandels auf den Tourismus: (i) direkte Auswirkungen eines veränderten Klimas (z. B. Hitze im Sommer, Extremereignisse, Abnahme der natürlichen Schneedecke), (ii) indirekte Auswirkungen von Umweltveränderungen (z. B. Verlust von Naturattraktionen), (iii) Klimapolitik und globale Tourismusmobilität (z. B. durch fiskalische Instrumente, die zu einem Anstieg der Reisekosten führen können) und (iv) gesellschaftlicher Wandel im Zusammenhang mit einem reduzierten Wirtschaftswachstum, veränderten Konsumverhalten und sozialpolitischer Stabilität.

Bisherige Forschungsarbeiten zum Einfluss des Klimawandels auf den Tourismus gehen davon aus, dass die in den nächsten Jahrzehnten zu erwartenden Veränderungen des Klimas sich sehr stark auf die österreichische Tourismusbranche auswirken werden (APCC 2014; Klima- und Energiefonds 2017). Die zu erwartenden Konsequenzen für den Tourismus werden jedoch sehr heterogen diskutiert. Dabei werden Auswirkungen nicht nur negativer, sondern auch positiver Art erwartet (Böhm 2008; Pröbstl-Haider et al. 2015). Die durch den Klimawandel bedingten monetären Verluste im Wintertourismus übersteigen allerdings noch immer die Zugewinne im Sommertourismus (Köberl et al. 2014). Der vorliegende *Austrian Special Report 2019* „Tourismus und Klimawandel" (ASR19) baut auf dem 2014 veröffentlichten Österreichischen Sachstandsbericht Klimawandel (AAR14 – Austrian Assessment Report 2014) auf (APCC 2014). Die damals bereits vorliegenden Studien werden hier validiert und durch neue Arbeiten ergänzt. Ziel ist es, eine umfassende Erhebung, Zusammenfassung und Bewertung des aktuellen Standes der Forschung zu den komplexen Beziehungen zwischen Tourismus und Klimawandel herzustellen.

Aus dem Zusammenspiel der regionalen und lokalen Unterschiede, die aufgrund der gegebenen Diversität, klimatischer Änderungen, der grundsätzlichen Klimasensibilität der jeweils angebotenen Tourismusaktivitäten und -attrak-

[1] $CO_2$-Äquivalente-Emission: Die Menge an $CO_2$-Emissionen, die über einen bestimmten Zeitraum denselben integrierten Strahlungsantrieb erzeugen würden wie die emittierte Menge des betrachteten Treibhausgases oder einer Mischung von Treibhausgasen. Die $CO_2$-Äquivalente-Emission stellt eine gebräuchliche Maßeinheit für den Vergleich von Emissionen unterschiedlicher Treibhausgase dar, impliziert jedoch keine Äquivalenz der entsprechenden Klimareaktion (IPCC 2013/2014).

tionen sowie der wirtschaftlichen Bedeutung des Tourismus zu erwarten sind, bedarf es auch differenzierter Anpassungsstrategien (vgl. APCC 2014). „Unter Anpassung werden Initiativen und Maßnahmen verstanden, die gesetzt werden, um ‚die Empfindlichkeit natürlicher oder menschlicher Systeme gegenüber tatsächlichen oder erwarteten Auswirkungen der Klimaänderung zu verringern' (IPCC 2007). Anpassungsaktivitäten zielen darauf ab, die Verwundbarkeit gegenüber der Klimaänderung zu reduzieren bzw. die Widerstandsfähigkeit (Resilienz) zu erhöhen sowie mögliche Chancen durch veränderte klimatische Bedingungen zu nutzen" (BMNT 2017, S. 26). Ansätze dazu liefern die jeweiligen Kapitel des vorliegenden Sachstandsberichts. Diese Überlegungen bilden die Grundlage für eine Zusammenschau der jeweiligen Möglichkeiten am Ende des Berichtes (Kap. 13).

Die Art der Anpassung kann in unterschiedlicher Form und auf verschiedenen Ebenen umgesetzt werden. Dabei lassen sich vorausschauende (proaktive) oder auf bestimmte Klimafolgen reagierende (reaktive) Maßnahmen unterscheiden. In diesem Bericht werden dabei alle Maßnahmen diskutiert, die, so weit möglich, geeignet sind die touristische Branche fit zu machen, sodass sie möglichst wenig durch die Auswirkungen des Klimawandels beeinträchtigt wird und diesen ihrerseits nicht weiter fördert. Anpassungsmaßnahmen schließen auch Strategien ein, die dazu dienen, sich die möglichen Folgen zunutze zu machen. Wichtig ist es, in diesem Zusammenhang auch eine mögliche Maladaptation hervorzuheben, also Anpassungsstrategien, die die negativen Effekte noch verstärken können, wie etwa Klimaanlagen (BMNT 2017).

Neben den Herausforderungen, denen sich der Tourismus in Österreich infolge der Auswirkungen des Klimawandels zu stellen hat, fällt dem Sektor als Mitverursacher des Klimawandels allerdings auch im Bereich der Minderung (Mitigation) Verantwortung zu. Aktuellen Untersuchungen zufolge trägt der Tourismus rund 8 % aller globalen $CO_2$-Emissionen und rund 5 % aller globalen $CO_2$-Äquivalent-Emissionen bei (Lenzen et al. 2018). Minderungsmaßnahmen, die eine aktive Verringerung der Treibhausgasemissionen zum Ziel haben, sind daher auch im Tourismus wichtig, um proaktiv zu einer Reduktion der Auswirkungen des Klimawandels beizutragen (vgl. BMNT 2017).

Der größte Anteil der touristischen Treibhausgasemissionen entfällt mit rund 50 % auf den Transport. Die An- und Abreise und Mobilität vor Ort sollten daher eine große Rolle bei Emissionsminderungsstrategien einnehmen (siehe Kap. 3 und 11; Lenzen et al. 2018). Es besteht jedoch auch ein erhebliches Potenzial zur Energie- und damit Emissionseinsparung in den Unterkünften und anderen touristischen Einrichtungen (Lund-Durlacher 2007; Formayer und Kromp-Kolb 2009; BMWFW et al. 2015), die größtenteils in den 1960er- und 1970er-Jahren gebaut wurden. Es gibt bereits zahlreiche effektive Praktiken zur Reduzierung des Energieverbrauchs[2] und der einhergehenden Emissionen. So können unter anderem Gebäudeisolierung und die Umstellung auf erneuerbare Energien wie Wind-, Solar- oder Geothermie die Nutzung fossiler Brennstoffe reduzieren, die zur Luftverschmutzung und zum Klimawandel beitragen (siehe Kap. 4). Neben entsprechenden technischen Maßnahmen kommt es zudem darauf an, in diesem Zusammenhang die Motivation von Mitarbeiterinnen, Mitarbeitern und Gästen zu beeinflussen. So lässt sich auch beim erholsamen Aufenthalt der Energieverbrauch noch reduzieren.

Neue Veröffentlichungen über nachhaltiges Hotelmanagement schlagen auch vor, den Beitrag von Lebensmittel- und Getränkekonsum zu den Treibhausgasemissionen stärker einzubeziehen. Rund 30–40 % des weltweiten Energieverbrauchs werden durch die Nahrungsmittelproduktion verursacht und die Art der Ernährung (z. B. der Fleischkonsum) hat einen direkten Einfluss auf die Höhe der Treibhausgasemissionen (FAO 2012). Einflussfaktoren wie die Produktionsprozesse der Lebensmittel, Transportdistanzen, Lagerung, Speisenzubereitung und Präsentation sowie das Verhalten der Gäste, insbesondere in Bezug auf Lebensmittelabfälle (Lund-Durlacher et al. 2016), sowie Minderungs- und Anpassungsstrategien für den Lebensmittel- und Getränkebereich können erhebliche Reduktionen der Treibhausgasemissionen erbringen (siehe Kap. 5).

Eine langfristige und nachhaltig positive Entwicklung des Tourismussektors lässt sich nur dann gewährleisten, wenn die vielfältigen Auswirkungen des Klimawandels rechtzeitig und richtig eingeschätzt werden sowie entsprechende Anpassungs- bzw. Vermeidungsstrategien angestoßen und umgesetzt werden. Dies ist die wesentliche Aufgabe des vorliegenden Berichts, der insbesondere die Tourismusbranche und politische Entscheidungstragende ansprechen soll, aber auch die Gäste auf ihre Eigenverantwortung in Hinblick auf ein möglichst klimaschonendes Verhalten aufmerksam machen will.

Dem vorliegenden Bericht liegt das Bewusstsein zugrunde, dass die Anforderungen durch den Klimawandel weit über bisherige Anstrengungen im Bereich der Nachhaltigkeit hinausgehen müssen. Mit dem Ziel „Netto Null 2050" geben IPCC, UNO und die Unterzeichner des Pariser Klimaabkommens klar vor, dass jede Branche gefordert ist, mit spezifischen Strategien dazu beizutragen. In Österreich soll dieses Ziel bis 2040 erreicht werden (Stand 2019). Für den Tourismus als Wirtschaftszweig, der sehr stark auf Mobilität, Beherbergung und Konsum beruht, ist das eine große Herausforderung.

[2] Korrekt wäre zwar der Begriff „Energiebedarf", da allerdings im Sprachgebrauch üblicherweise von „Energieverbrauch" die Rede ist, wird dieser Begriff auch im Rahmen des vorliegenden Berichts so verwendet.

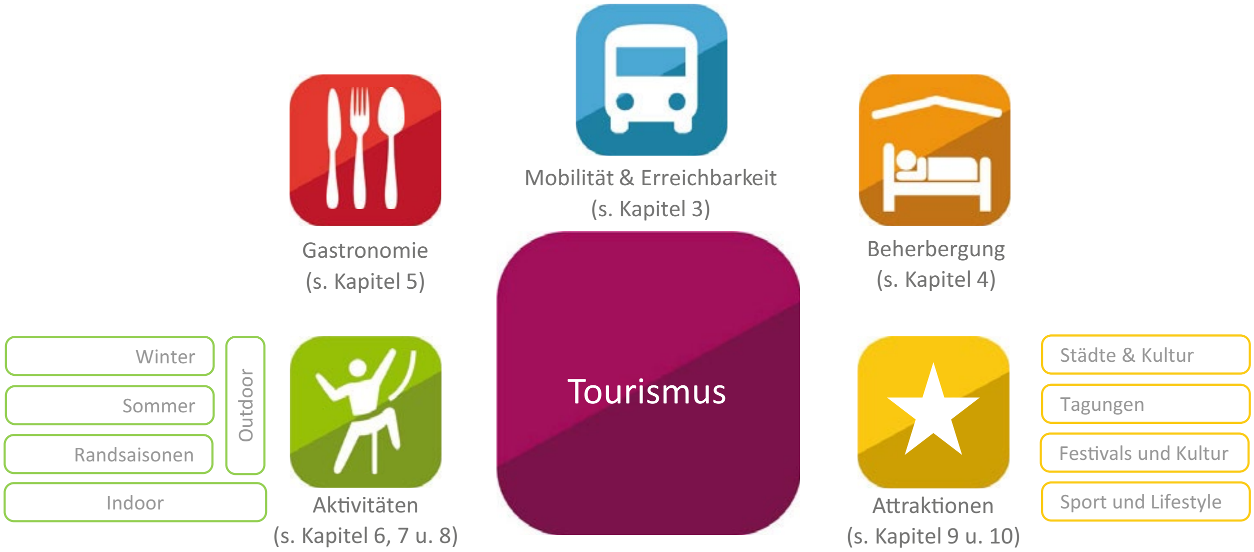

**Abb. 1.1** Wichtige Bausteine des Tourismussystems, die im Zusammenhang mit dem Klimawandel zu betrachten sind, mit Verweisen auf die entsprechenden Kapitel im vorliegenden Bericht. (Basierend auf Dickman 1997; Cooper 2008; Getz und Page 2016; Grafik: Ulrike Pröbstl-Haider und Thomas Schauppenlehner)

### 1.1.1 Entstehungsprozess

Der ASR19 wurde in einem umfassenden Teamwork unterschiedlichster Forschungseinheiten zusammengestellt und einem kritischen Review von nationalen und internationalen deutschsprachigen Gutachterinnen und Gutachtern unterzogen. Das Konsortium umfasst das Spektrum von führenden Forschungsinstituten in diesem Bereich und liefert einen umfassenden Einblick in die wissenschaftliche Arbeit der in diesem Bereich tätigen Personen. Mehrere Autorenworkshops erleichterten die Koordination und Kommunikation während der Durchführung des Projekts. Durch zwei Fachtagungen und direkte Konsultationen wurde auch das Expertenwissen bestmöglich in den Bericht integriert.

Insgesamt waren 39 Autorinnen und Autoren an der Erstellung des ASR19 beteiligt, unter der Leitung von Ulrike Pröbstl-Haider (Universität für Bodenkultur) und der drei weiteren Co-Chairs Dagmar Lund-Durlacher (Modul University Vienna), Marc Olefs (Zentralanstalt für Meteorologie und Geodynamik) und Franz Prettenthaler (Joanneum Research Forschungsgesellschaft mbH). Die koordinierenden Leitautorinnen und -autoren (CLA – Coordinating Lead Authors) waren jeweils für ihr Kapitel sowie die Koordination der Leitautorinnen und -autoren (LA – Lead Authors) und beitragenden Autorinnen und Autoren (CA – Contributing Authors) verantwortlich. LAs trugen wesentlich zu ihren jeweiligen Kapiteln bei, etwa mit Subkapiteln, und sind gemeinsam mit den CLAs für das gesamte Kapitel inhaltlich verantwortlich. CAs steuerten ohne gesamtinhaltliche Verantwortung für das Kapitel kleinere Teile, Materialien oder thematisch relevante Fachbeiträge bei, die von den CLAs bzw. LAs in die jeweiligen Kapitel integriert wurden.

Der Reviewprozess, koordiniert vom CCCA, erfolgte in mehreren Stufen. Der Zero-Order-Draft mit der Grundstruktur des Berichts wurde einem informellen, internen Review unterzogen. Der erste inhaltliche Entwurf (First-Order-Draft) erging in einem ersten externen Review an 16 nationale und internationale Gutachterinnen und Gutachter. Auf Basis der Reviewkommentare wurde der erste Entwurf überarbeitet und einer zweiten Reviewschleife (19 Gutachterinnen und Gutachter) unterzogen. In einer weiteren Überarbeitung wurde der Final Report (Draft) erstellt. Die Reviewkommentare wurden aufgegriffen oder mit einer erklärenden Begründung abgelehnt. Die Einarbeitung der Kommentare wurde von 14 Revieweditoren überprüft. Eine finale Überarbeitung erfolgte auf Basis des Feedbacks der Revieweditoren, unterstützt durch zusätzliche Anmerkungen des APCC.

### 1.1.2 Aufbau und Inhalt des Berichts

In Kap. 2 werden die grundlegenden klimatologischen Rahmenbedingungen für den Tourismus in Österreich dargelegt. Es wird dabei sowohl auf das vergangene Klima als auch auf das zukünftige Klima eingegangen und die Klimawandelfolgen werden sowohl anhand klassischer meteorologischer als auch mithilfe tourismusspezifischer Indikatoren (z. B. Tourism Climate Index (TCI), Mieczkowski 1985) aufgezeigt.

Die Auseinandersetzung mit dem komplexen Verbundsystem Tourismus erfolgt im vorliegenden Bericht so umfassend wie möglich. Das bedeutet, dass mehr als bisher auch die einzelnen Elemente eines touristischen Produktes von der Anreise über die Übernachtung, die Aktivitäten bis hin zur Gastronomie miteinbezogen werden. Hier liefert der vorliegende Bericht erstmalig einen differenzierten Zugang. Die Struktur des Tourismussystems, die auch diesem Bericht zugrunde liegt, ist in Abb. 1.1 dargestellt. Sie zeigt die entscheidenden Bausteine des Tourismussystems (blau dargestellt), die hier im Zusammenhang mit dem Klimawandel detailliert betrachtet werden, und die für das Reisemotiv ganz entscheidenden Attraktionen und Aktivitäten (hier orange dargestellt), jeweils mit Verweisen auf die entsprechenden thematischen Kapitel des Berichts. Allerdings sind wir uns bewusst, dass für das touristische Erlebnis diese „Bausteine" zusammenwirken und oft vom Gast als ganzheitlich wahrgenommen werden. Die separate Betrachtung erleichtert jedoch die Analyse und Ableitung von Handlungsoptionen.

Der vorliegende Bericht stellt für die jeweiligen Bereiche neben dem Kernthema des Einflusses des Klimawandels bzw. auf den Klimawandel auch die aktuellen Trends und Entwicklungen dar, um mögliche Veränderungen auf der Angebots- und Nachfrageseite nicht zu übersehen bzw. diese bei der Diskussion von Anpassungsmaßnahmen und Minderungsstrategien berücksichtigen zu können. Dabei werden die klassischen Komponenten eines Urlaubs, die Mobilität, der Transport und die Erreichbarkeit von Destinationen (Kap. 3), die Beherbergung (Kap. 4), die Gastronomie und Kulinarik (Kap. 5) sowie die häufig urlaubsprägenden Aktivitäten und Attraktionen jeweils gesondert betrachtet. Die Aktivitäten gliedern sich im Bericht in Outdooraktivitäten im Winter (Kap. 6), Outdooraktivitäten im Sommer und in den Übergangszeiten (Kap. 7) sowie Indooraktivitäten (Kap. 8).

Die ausführliche Behandlung der Attraktionen, zu denen hier auch der Städte- und Kulturtourismus (Kap. 9), Festivals, kulturelle Events, Großveranstaltungen sowie Sportgroßveranstaltungen und Lifestyleevents (Kap. 10) zählen, fehlte im letzten Sachstandsbericht (AAR14). Viele der Marketinginitiativen, wie Kultur- oder Sportveranstaltungen, die viele Besucherinnen und Besucher anziehen und erhebliche Treibhausgasemissionen verursachen können, wurden ebenfalls in den Bericht aufgenommen. Die Berücksichtigung dieser Ereignisse muss auch die möglichen Auswirkungen des Klimawandels auf verschiedene Arten von Veranstaltungen, ihre Besucherinnen und Besucher und die Möglichkeiten zur Minderung und Anpassung einschließen.

Kap. 11 greift die globale Dimension des Tourismus auf und diskutiert einerseits die aktuellen Erkenntnisse des Beitrags des Tourismus zu den weltweiten Treibhausgasemissionen und andererseits die klimawandelinduzierten regionalen Verschiebungen der Tourismusnachfrage und deren Auswirkungen auf Österreich. Da es bislang keine detaillierten Studien zum Beitrag des Tourismus zu den Treibhausgasemissionen für Österreich gibt, wurden aufgrund der Bedeutung für Minderungsstrategien erste grobe Abschätzungen zumindest für die An- und Abreise sowohl für den Tourismus aus Österreich (österreichische Reisende, auch ins Ausland) als auch für den Tourismus in Österreich (In- und Ausländer) gemacht.

Nationale Verpflichtungen auf Grundlage des Pariser Klimaabkommens und Strategiepapiere werden in Kap. 12 diskutiert, bevor abschließend in Kap. 13 mögliche Handlungsoptionen für alle Bereiche zusammenfassend aufgezeigt werden.

Der folgende einführende Abschn. 1.2 erläutert die Bedeutung des Tourismus in Österreich und liefert damit wichtige Hintergrundinformation zum Verständnis der jeweiligen Fachkapitel, die die einzelnen Bereiche des Tourismus im Zusammenhang mit dem Klimawandel vertiefend betrachten.

## 1.2 Stellenwert und Entwicklung des Tourismus in Österreich

Wenn nachstehend die gesamtwirtschaftliche Bedeutung des Tourismus behandelt wird, dann ist es nötig zu wissen, dass im Rahmen der Tourismusstatistik in Österreich die Reiseaktivitäten von Personen erfasst werden, die zumindest eine Nächtigung außer Haus in Österreich umfassen, in der Freizeit erfolgen und keinen beruflichen, gesundheitlichen o. ä. Anlass haben. Alle österreichischen Beherbergungsbetriebe und Privatvermietungen sind durch die bundesweite Tourismus-Verordnung von 2002 verpflichtet monatlich entsprechende Daten an ihre Gemeinde zu melden. Die Datenerfassung entspricht damit den internationalen Anforderungen (UN und UNWTO 2010).[3]

### 1.2.1 Gesamtwirtschaftliche Bedeutung

Betrachtet man die touristische Entwicklung Österreichs in Zahlen, dann zeigt sich nicht nur die enorme Bedeutung des Tourismus, sondern auch die hohe Wettbewerbsfähigkeit des Landes im internationalen Vergleich. Im Kalenderjahr 2018 wurden in Österreich über 44,8 Mio. Gäste mit rund 149,80 Mio. Übernachtungen gezählt, was einem erneuten Spitzenwert entspricht (Statistik Austria 2019a). Der Travel & Tourism Competitiveness Report 2019 unterstreicht die dargestellten Ergebnisse (WEF 2019). Die wirtschaftliche Bedeutung des Tourismus lässt sich auch an folgenden Kennzahlen gut nachvollziehen:

Von 2000 bis 2017 ist die touristische Gesamtnachfrage im Durchschnitt um 3,3 % pro Jahr gestiegen. Im Jahr 2017 beliefen sich die direkten Wertschöpfungseffekte des Tourismus – exklusive Dienst- und Geschäftsreisen – laut Tourismus-Satellitenkonto (TSA) auf 23,61 Mrd. €, was einem Anteil von 6,4 % am Bruttoinlandsprodukt entspricht. Für das Jahr 2018

[3] Bei vielen Daten zur Reisetätigkeit können berufliche Fahrten bzw. Konferenzteilnahmen nicht herausgerechnet bzw. ausgeklammert werden.

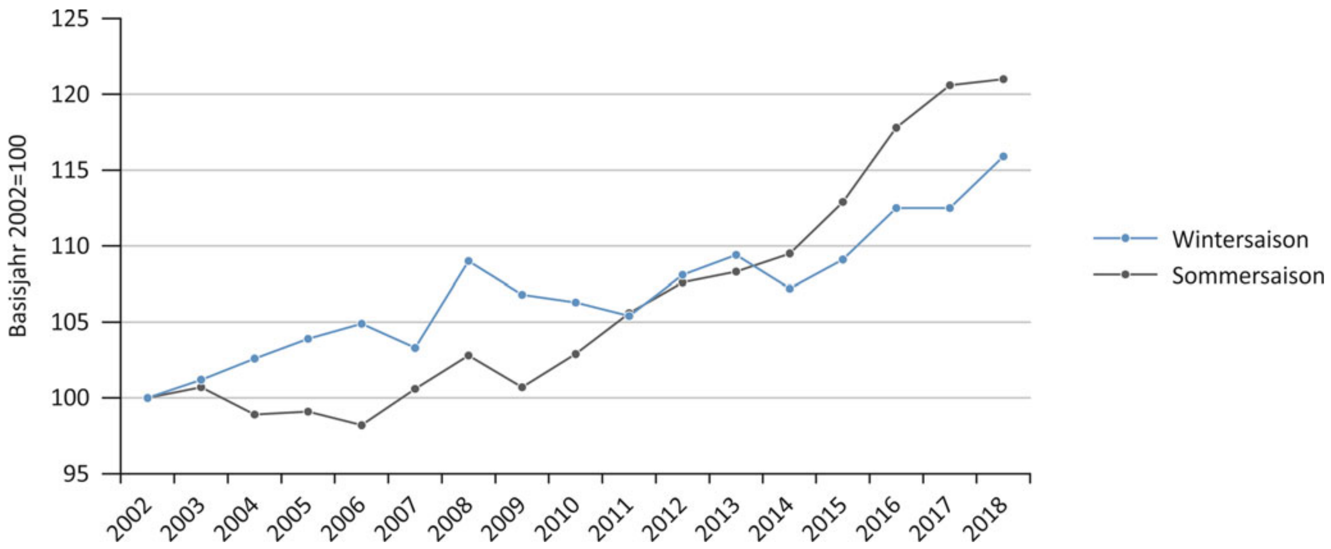

**Abb. 1.2** Übernachtungen laut Beherbergungsstatistik: Die touristische Entwicklung ist im letzten Jahrzehnt durch einen deutlichen Anstieg gekennzeichnet, wobei die Sommersaison (01.05.–31.10.) zuletzt stärkere Anstiege bei den Übernachtungen verzeichnet als die Wintersaison (01.11.–30.04.). (Indexdarstellung 2002 = 100 %; Statistik Austria 2019a) (Quelle: Statistik Austria, Tourismus - Beherbergungsstatistik 2018)

wird von einer Steigerung auf ca. 25 Mrd. € und einem Anteil von 6,5 % am BIP ausgegangen. Bezieht man zusätzlich auch die indirekten Wertschöpfungseffekte des Tourismus mit ein, so belaufen sich die für 2018 angenommenen Werte sogar auf 32,25 Mrd. € und 8,4 % des BIP (Fritz et al. 2019; Statistik Austria 2019b). Aufgrund seiner großen Bedeutung und der damit einhergehenden Beschäftigungseffekte leistet der Tourismus in Österreich einen wesentlichen Beitrag zur Gesamtbeschäftigung. Laut TSA-Beschäftigungsmodul betrug das direkte touristische Beschäftigungsvolumen im Jahr 2017 rund 317.500 Arbeitsverhältnisse bzw. 244.000 Vollzeitäquivalente; Letzteres entspricht einem Anteil von 6,4 % der bundesweiten Gesamtbeschäftigung (Fritz et al. 2019)[4].

Sowohl der Tourismus im Sommer als auch im Winter zeigen einen deutlichen Anstieg gemessen an der Zahl der Übernachtungen. Stärkere Zuwächse waren zuletzt in den Sommermonaten zu erkennen (vgl. Abb. 1.2).

Eine Analyse der erzielten Wertschöpfung im Vergleich zeigt die besondere Bedeutung des Winterurlaubs. Die durchschnittlichen Tagesausgaben (Unterkunft, Verpflegung, Transportkosten, ohne An- und Rückreise) in Österreich im Tourismusjahr 2018/2019 betrugen im Winter 185 €, im Sommer 2018 wurden demgegenüber nur 160 € ausgegeben (Österreich Werbung 2018, 2019a). Zu beachten sind zudem die verschiedenen Kategorien der Urlaubsarten, wonach z. B. der Wintersporturlauber im Vergleich zum Wander-/Bergsteigurlauber rund 43 € mehr ausgibt (inkl. Mobilität; s. Abb. 1.4). Diese Unterschiede spiegeln sich dann in den Umsätzen wider, die im Winter deutlich höher ausfallen. Trotz höherer Übernachtungszahlen im Sommer, werden in den Wintermonaten höhere Umsätze erzielt. Im Jahr 2017 betrug die Differenz 0,5 Mrd. €, im Jahr 2018 sogar 0,8 Mrd. €. Die absoluten Zahlen sind in Abb. 1.3 wiedergegeben.

Eine negative Entwicklung im Wintertourismus aufgrund des Klimawandels würde daher manche Tourismusdestinationen besonders stark treffen, da der Anteil der im Beherbergungs- und Gaststättenwesen erwerbstätigen Personen teilweise über 80 % an den Gesamterwerbstätigen einnimmt (Prettenthaler und Formayer 2011).

### 1.2.2 Herkunftsländer des österreichischen Tourismus

Die wichtigsten Herkunftsländer sind neben dem Inlandstourismus vor allem Deutschland, die Niederlande und Schweiz/Liechtenstein (s. Abb. 1.5). Daher wird in den spezifischen Kapiteln vor allem auf die Entwicklung in den wichtigsten Herkunftsländern, insbesondere Deutschland, eingegangen. Insgesamt ist die Anzahl an Nächtigungen im Vergleich 2017/2018 um 3,7 % gestiegen, der größte Zuwachs kommt aus Deutschland mit 5,1 %. Die Nachfrage aus der Schweiz/Liechtenstein ist um 1,7 % gesunken. Urlauberinnen und Urlauber aus EU-28-Ländern tragen einen Anteil von 88,3 % (132,3 Mio. Übernachtungen) an den Gesamtübernachtungen bei. Wie Abb. 1.5 zeigt, ist der Inlandsurlaub in Österreich ebenfalls ein wichtiges Urlaubersegment.

[4] Mit Stand Januar 2020 liegen noch keine aktuelleren Zahlen zum direkten touristischen Beschäftigungsvolumen vor, daher können an dieser Stelle nur die Daten aus 2017 angeführt werden.

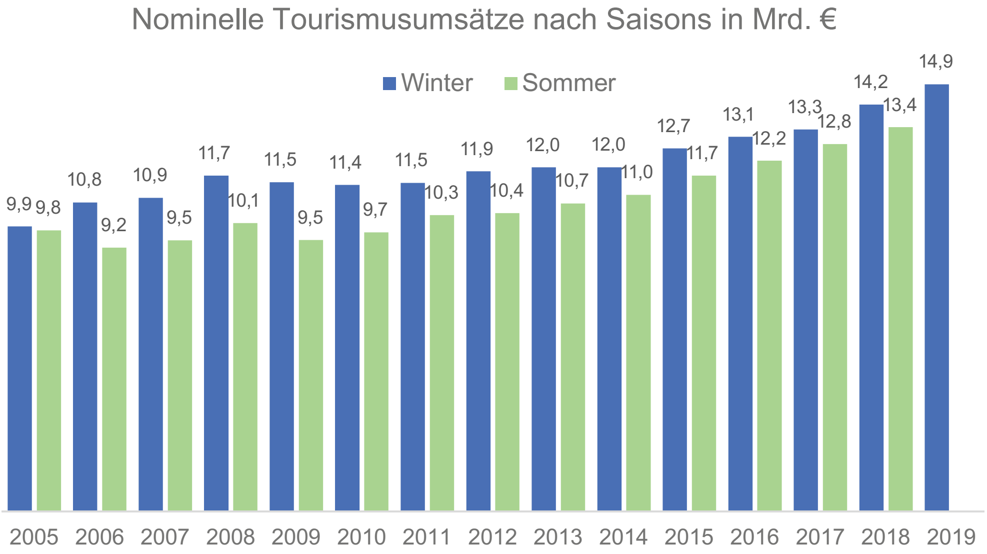

**Abb. 1.3** Nominelle Tourismusumsätze nach Saisonen für Österreich, im Winter werden höhere Umsätze erzielt. Dabei wird der Winter als Zeitraum 01.11.–30.04. und der Sommer mit 01.05.–31.10. definiert. (Datenquelle: WIFO 2019; Grafik: Magdalena Feilhammer)

### 1.2.3 Zeitliche und saisonbezogene Verteilung

Die langfristige Entwicklung der Übernachtungszahlen sowohl im Sommerhalbjahr als auch im Winterhalbjahr sind beide durch einen Anstieg gekennzeichnet. Interessant ist, dass sich in den letzten Jahrzehnten ein schrittweiser Rückgang des Sommertourismus zeigte, während die Übernachtungszahlen im Winter beständig anstiegen und schließlich ab 2003 im Umfang den Sommer in einzelnen Jahren sogar überstiegen. Seit 2014 hat sich das Verhältnis wieder umgekehrt und die Nächtigungszahlen in den Sommermonaten liegen wieder etwas höher (s. Abb. 1.6). Dabei hat in den letzten Jahren die Frühjahrs- und Herbstsaison an Bedeutung gewonnen (s. Abb. 1.7).

### 1.2.4 Räumliche Verteilung touristisch intensiv genutzter Regionen

Die touristische Nutzung ist in Österreich durch eine starke räumliche Konzentration charakterisiert. In touristisch intensiv genutzten Gemeinden kommen pro Tourismusjahr bis zu über 1000 Nächtigungen auf eine Einwohnerin bzw. einen Einwohner (Prettenthaler und Formayer 2011). Die tourismusintensivsten Gemeinden mit einer hohen Nächtigungsanzahl sind v. a. in Tirol und Salzburg zu finden (s. Abb. 1.8). Auf Tirol entfielen im Jahr 2018 rund 33 % und auf Salzburg 19,7 % der Nächtigungsanteile (Statistik Austria 2019a). Bei detaillierter Betrachtung werden auch die großen Disparitäten auf Gemeindeebene deutlich (Tappeiner et al. 2008; Price et al. 2011). In den in Abb. 1.8 rot dargestellten tourismusintensiven Gemeinden kann dies Überlastungen der kommunalen Infrastruktur, Gentrifizierung und Beeinträchtigungen des sozialen Lebens, insbesondere in ländlichen Räumen, bedeuten.

Daneben zeigen sich auch in der räumlichen Verteilung große Unterschiede zwischen den österreichischen Regionen, wenn man Sommer und Winter getrennt betrachtet. So sind die Sommermonate – wie in Abb. 1.9 dargestellt – im Burgenland, in Kärnten, Nieder- und Oberösterreich durch eine hohe Bettenauslastung gekennzeichnet, während für Salzburg, Tirol und Vorarlberg der Winter die meisten Gäste anzieht. Der Städtetourismus in Wien ist auch in den Sommermonaten auffällig hoch. Die Steiermark mit sehr heterogenen Strukturen (besondere Einrichtungen für den Wintersport im Norden, sommertouristische Ausrichtung im Süden) nimmt insgesamt eine mittlere Position ein (s. Abb. 1.9).

### 1.2.5 Aufenthaltsdauer

Im Hinblick auf die Aufenthaltsdauer der Touristinnen und Touristen in Österreich läuft der derzeitige Trend im Urlaubsverhalten – anstelle einer klimaschonenden Verlängerung der Aufenthaltsdauer bei gleichzeitiger Verringerung der Anzahl der Reisen – in die entgegengesetzte Richtung. Seit Jahren nimmt die durchschnittliche Aufenthaltsdauer der Gäste kontinuierlich ab. Während sie beispielsweise im Jahr 1987 noch bei 5,3 Tagen lag, betrug sie im Jahr 2017 nur noch 3,4 Tage (s. Abb. 1.10); im darauffolgenden Jahr 2018 lag der Durchschnittswert nur mehr bei 3,3 Tagen (Statistik Austria 2019a). Da die Tendenz zu einer kürzeren Aufenthaltsdauer besteht, die nicht nur in Österreich, sondern weltweit zu beobachten ist, wird eine Umkehrung dieses Trends derzeit als nicht wahrscheinlich erachtet.

Bei genauerer Betrachtung zeigen sich jedoch erhebliche Unterschiede zwischen den österreichischen Regionen und Bundesländern (s. Abb. 1.10).

Insbesondere in Bundesländern wie Wien, Ober- und Niederösterreich, die durch intensiven Städte- und Tagungs-

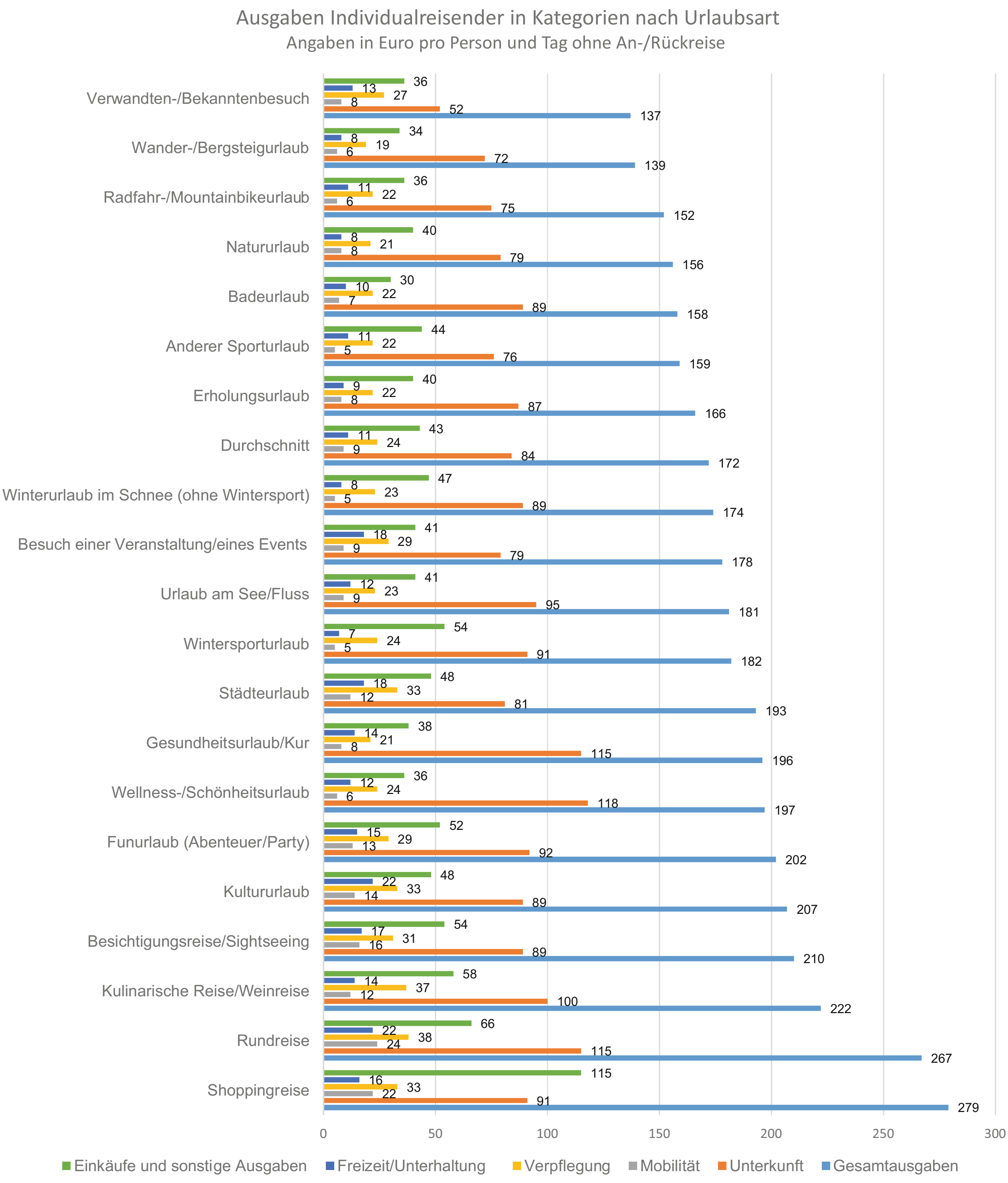

**Abb. 1.4** Ausgaben von Individualreisenden nach Urlaubskategorie und Ausgabenort, gerundete Werte, $n$ = rund 30.300. (Datenquelle: Österreich Werbung 2018, 2019a; Grafik: Magdalena Feilhammer)

**Abb. 1.5** Angabe der jährlichen Nächtigungen in Österreich 2018 nach wichtigen Herkunftsländern, im Vergleich 2017 zu 2018. (Datenquelle: Statistik Austria 2019c; Grafik: Magdalena Feilhammer)

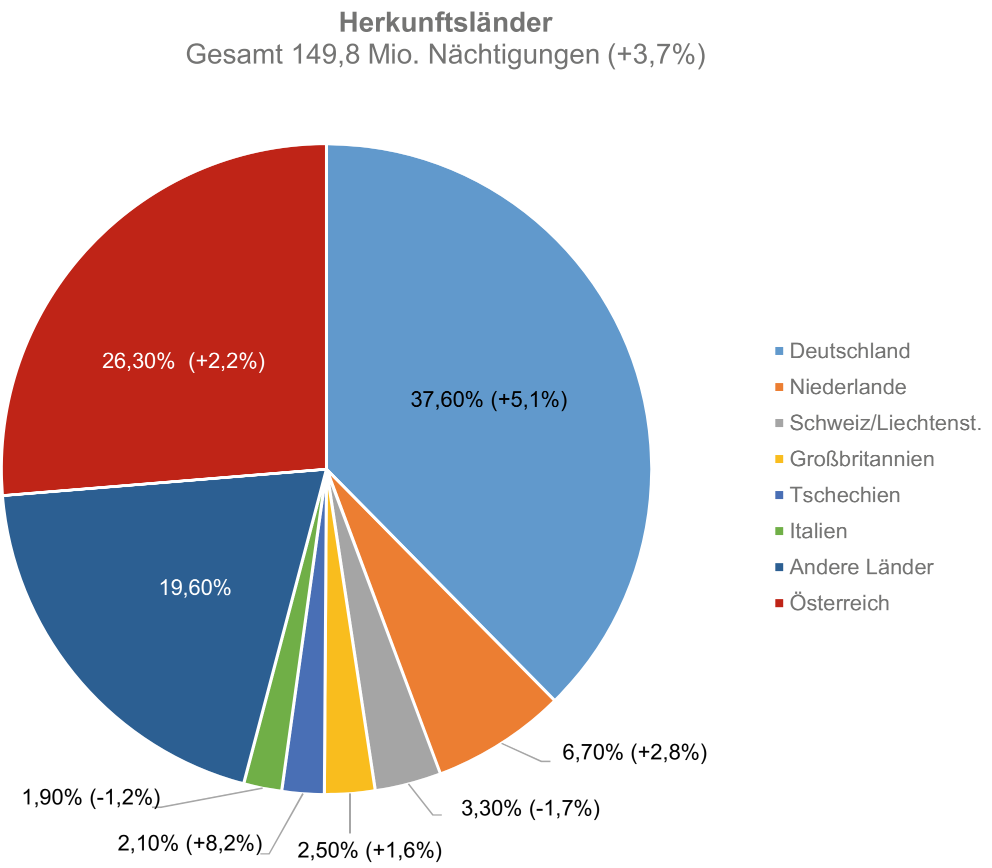

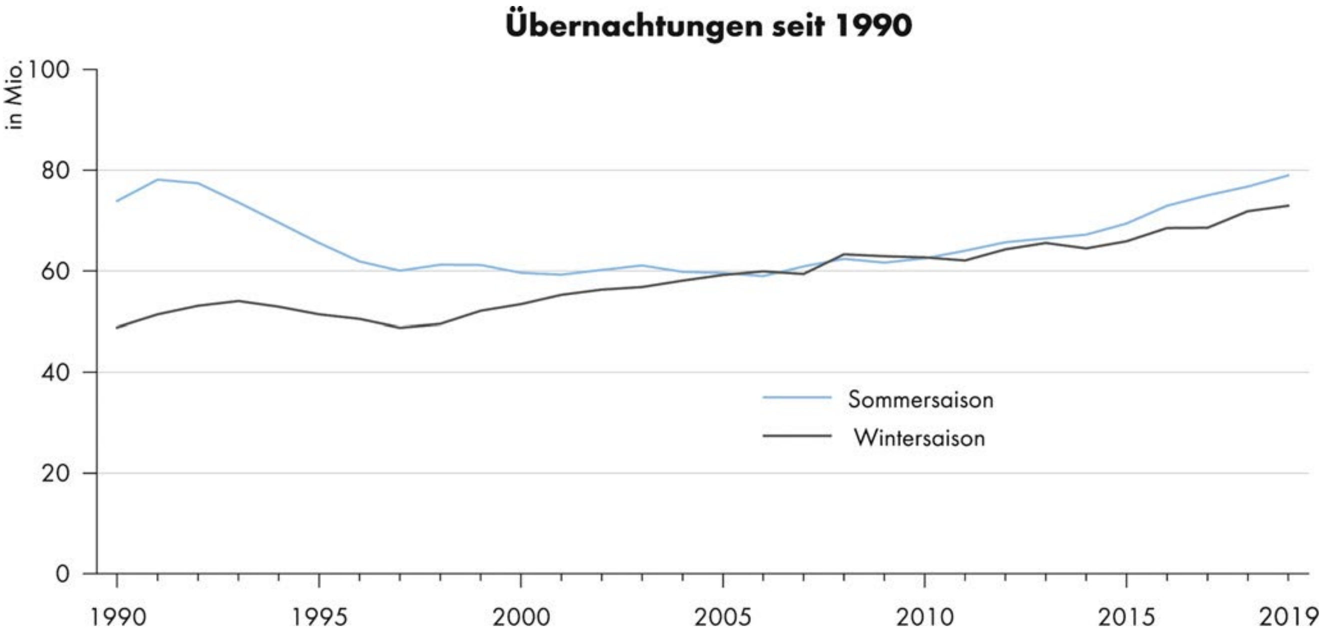

**Abb. 1.6** Übernachtungen für Österreich seit 1990. (Statistik Austria 2020a) (Quelle: Statistik Austria, Tourismusstatistik. Erstellt am 04.03.2020.)

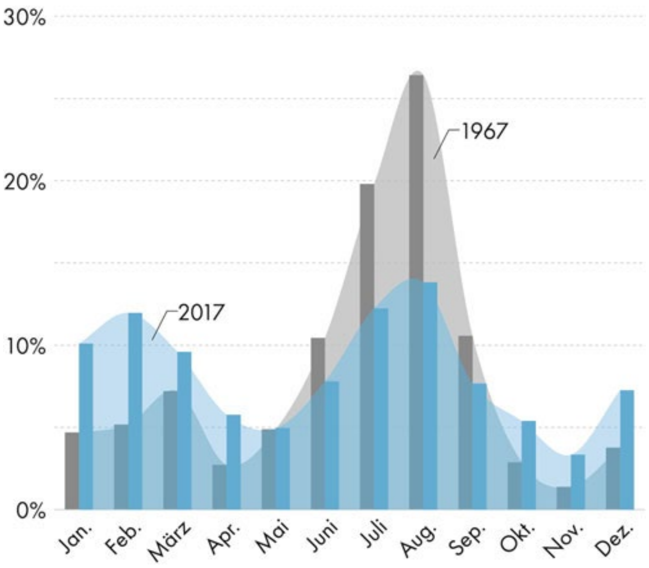

**Abb. 1.7** Der Vergleich zwischen der Saisonalität (Jahresgang der Nächtigungen mit monatlichen Anteilen in Prozent) zwischen den Werten von 1967 (*grau*) und 2017 (*blau*) für Österreich zeigt, dass sich die saisonale Verteilung deutlich verändert hat. (Statistik Austria 2018; Darstellung verändert)

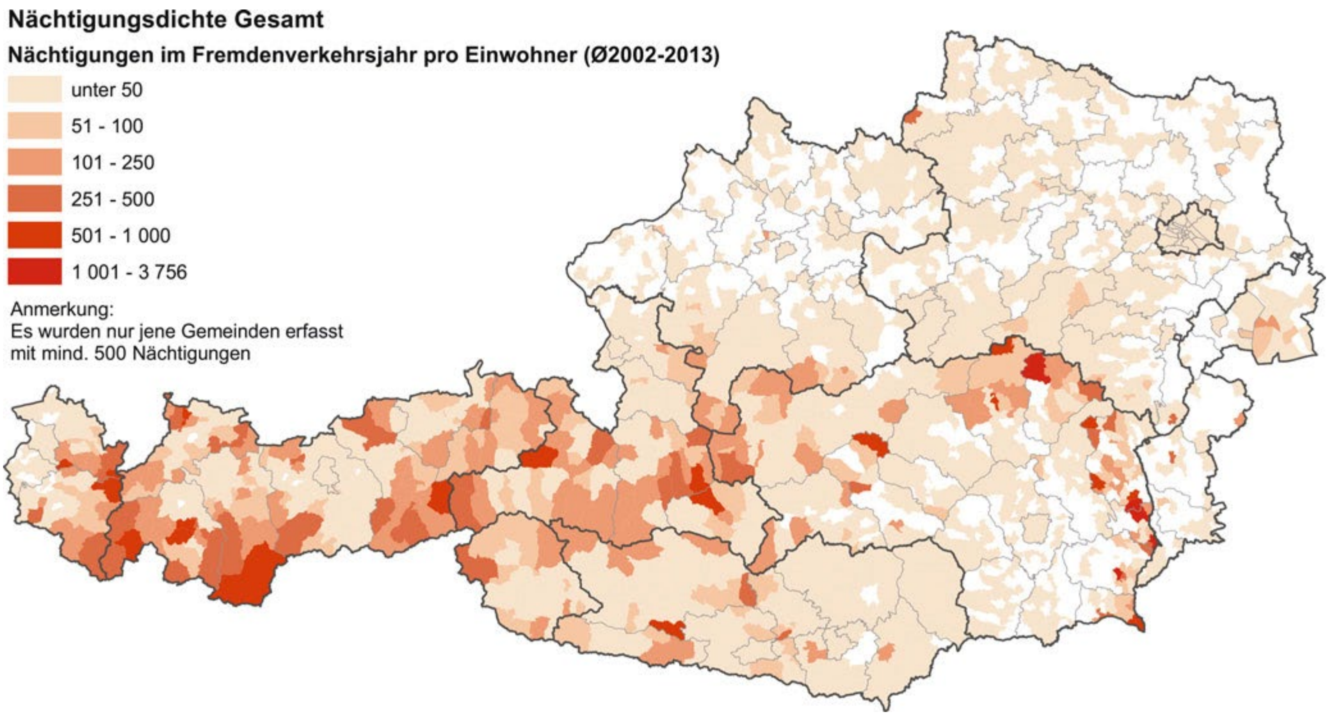

**Abb. 1.8** Nächtigungsdichte (Nächtigung pro Einwohner und Jahr dargestellt auf Gemeindeebene). (Prettenthaler und Formayer 2011)

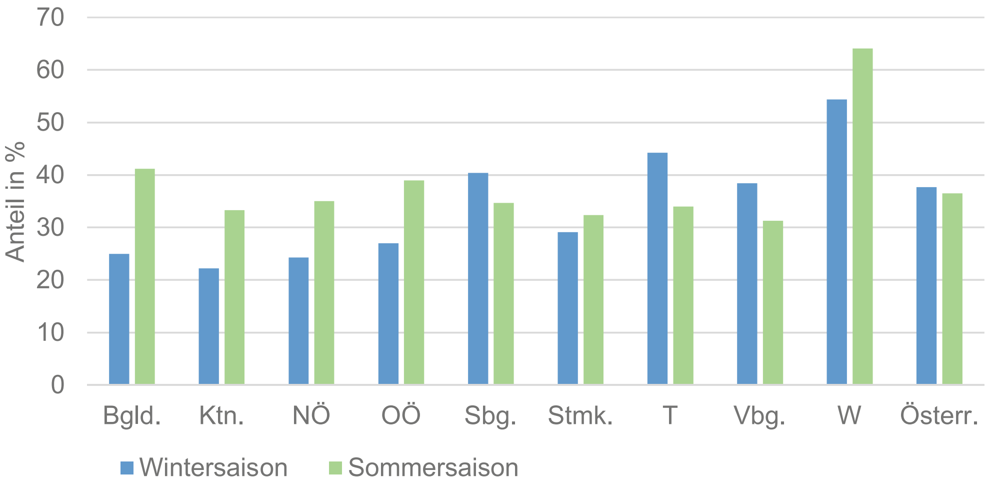

**Abb. 1.9** Bettenauslastung in der Wintersaison 2018/2019 (01.11.–30.04.) und Sommersaison 2019 (01.05.–31.10.) nach Bundesländern (Burgenland, Kärnten, Niederösterreich, Oberösterreich, Salzburg, Steiermark, Tirol, Vorarlberg und Wien). (Datenquelle: Statistik Austria 2020b; Grafik: Magdalena Feilhammer)

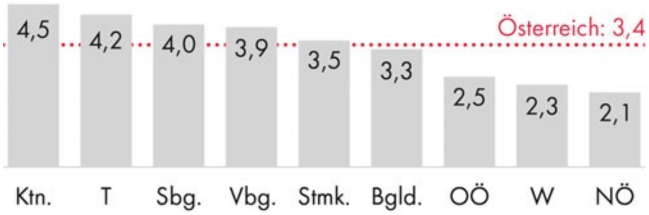

**Abb. 1.10** Durchschnittliche Aufenthaltsdauer der Gäste pro Reise und Person je Bundesland (Kärnten, Tirol, Salzburg, Vorarlberg, Steiermark, Burgenland, Oberösterreich, Wien und Niederösterreich) und für Österreich insgesamt. (Statistik Austria 2018; Darstellung verändert)

tourismus gekennzeichnet sind, ist die Aufenthaltsdauer deutlich kürzer.

### 1.2.6 Reisemotive

Die Reisemotive der Gäste sind neben persönlich motivierten Reisen vor allem durch einen Aktivurlaub gekennzeichnet. Erst danach folgen Motive mit Bezug zu Erholung und Entspannung.

Die Entscheidungsgründe für einen Urlaub in Österreich unterscheiden sich je nach Saison. Laut einer Umfrage der Österreich Werbung (2019b) locken im Winter besonders die Attraktivität der Skigebiete, das Wintersportangebot, Hotels und Unterkünfte, die Schneesicherheit auf den Pisten und die Berge die Gäste nach Österreich (s. Abb. 1.11). Im Sommer sind vor allem die Berge, die Landschaft und Natur sowie das Angebot an Wanderwegen ausschlaggebend, gefolgt von Seen und Flüssen (Österreich Werbung 2019c; s. Abb. 1.12).

### 1.2.7 Touristische Trends

Die Beschäftigung mit gesellschaftlichen Trends im Zusammenhang mit Klimawandel und Tourismus ist in zweierlei Hinsicht wichtig. Zum einen erlaubt die Trendforschung gewisse Entwicklungen im Konsumverhalten vorauszusehen und Anpassungsstrategien auf diese Entwicklungen hin abzustimmen. Zum anderen liefert die Trendforschung auch Anhaltspunkte für eine veränderte Produktentwicklung und Möglichkeiten, einen veränderten Lebensstil zu fördern, der zur Vermeidung und Verminderung von Treibhausgasen beiträgt, ohne dass dies dem Kunden bewusst ist. Diese Form der Einflussnahme wird in der Literatur als Nudging (schubsen, anstoßen) bezeichnet und wird als eine besondere Form der Governance, aber auch einer speziellen „Entscheidungsarchitektur" eingestuft (Thaler und Sunstein 2008; Baldwin et al. 2011; Levi-Faur 2011; Bradbury et al. 2013; Hall 2013; Kosters und Van der Heijden 2015).

Aus der großen Anzahl und Vielgestaltigkeit der diskutierten Trends werden nachstehend wichtige von verschiedenen Autorinnen und Autoren beschriebene Entwicklungen zusammengefasst (vgl. auch Bandi Tanner und Müller 2019).

#### Gesundheitsbezogener Tourismus, Health-Empowerment, Convenience und Mood-Management

Ein wichtiger Trend hängt mit der älter werdenden Gesellschaft zusammen, basiert aber auch auf einer Arbeits- bzw. Alltagssituation, die von vielen Menschen als belastend und sehr anstrengend beschrieben wird. Dabei geht es nicht nur um körperliche Fitness, sondern auch um seelische Entspannung und ein allgemeines Wohlbefinden. Dabei verwischen sich die Grenzen zwischen gesundheitsfördernden Maßnahmen, Erholung und Genuss. Prävention und die Förderung eines langfristigen gesundheitsfördernden Lebensstiles gewinnen dabei zunehmend an Bedeutung. Dies wird auch im Zusam-

**Abb. 1.11** Entscheidungsgründe für einen Winterurlaub in Österreich. (Österreich Werbung 2019b)

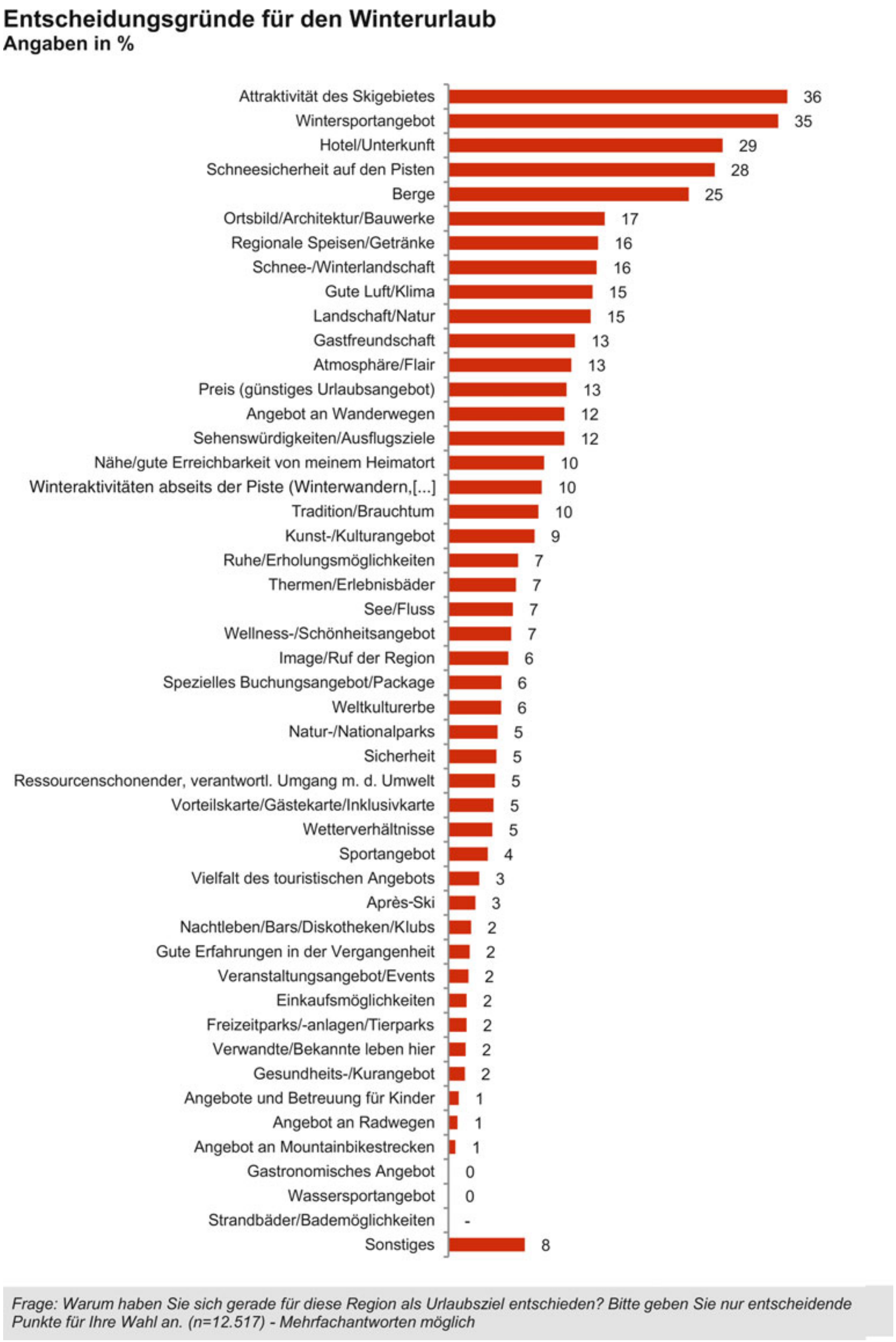

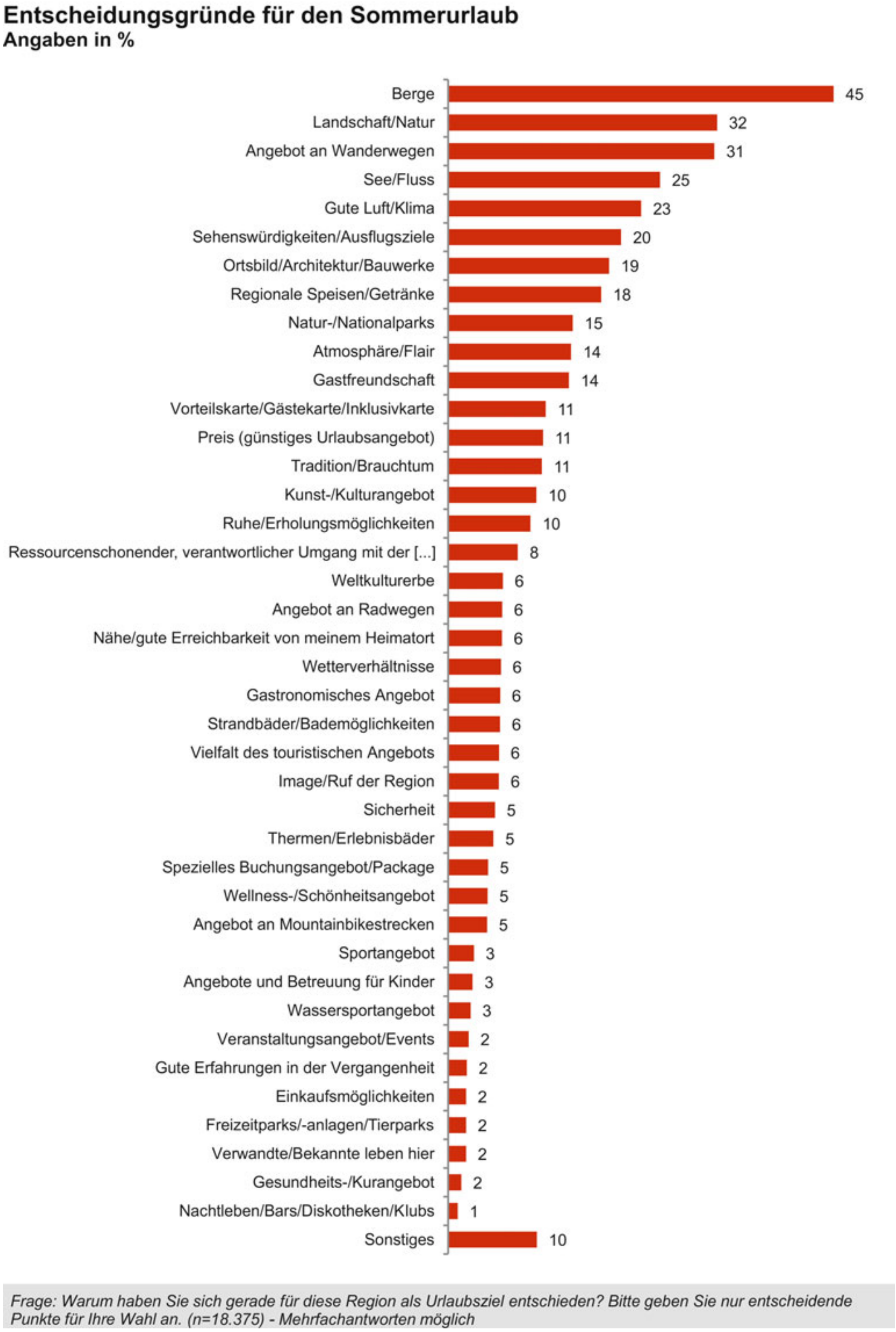

**Abb. 1.12** Entscheidungsgründe für einen Sommerurlaub in Österreich. (Österreich Werbung 2019c)

menhang mit einem „Luxus", den man sich für einige wenige Tage gönnt, verbunden (Zellmann und Mayrhofer 2015).

In engem Zusammenhang mit der älter werdenden Gesellschaft, aber auch mit der kurzen Aufenthaltsdauer sind die Suche nach und Präferenz für Angebote zu sehen, die eine hohe Bequemlichkeit versprechen. Zeit ist ein begrenzender Faktor, der nicht mit beispielsweise mühsamer Anreise „vertan" werden soll. Trendforscher weisen darauf hin, dass extravaganter Service und ausgefallene Leistungen nicht nur von Besserverdienern erwartet und beansprucht werden, sondern dass auch die breiten Gästekreise immer stärker nach „Deep-Support-Lösungen" verlangen (Wenzel und Kirig 2006; Bandi Tanner und Müller 2019). Hohe Qualität im Service, Emotionen und Empathie der Mitarbeiterinnen und Mitarbeiter werden immer wichtiger, also Kommunikationskompetenz und Konfliktmanagement. In diesem Zusammenhang geht es um das allgemeine Wohlfühlen, auch als „Mood-Management“ bezeichnet. Dabei werden – dies gilt gerade für reiseerfahrene ältere Personen oder Personen im Management mit viel Reisetätigkeit – nicht länger die Urlaubsziele, sondern die Befriedigung des Wunsches nach Service, Convenience sowie nach ganzheitlichen Wohlfühlkonzepten ausschlaggebend. Dieser Aspekt könnte ein wichtiger Ansatzpunkt für Vermeidungsmaßnahmen sein, wenn dadurch z. B. der Inlandstourismus mit bequemer Anreise gefördert werden könnte (Schuckert und Müller 2006; Kästle 2012). Auf der anderen Seite ist diese Urlaubsform durch Kurzreisen gekennzeichnet, die auch mehrfach im Jahr durchgeführt werden. Dies ist im Hinblick auf die Treibhausgasemissionen ungünstig.

### Individualisierung

Ein weiterer wichtiger Trend wird unter dem Begriff „Individualisierung“ zusammengefasst. Dieser Begriff steht in der Gesellschaft für einen reduzierteren Wertekonsens und eine Wertepluralität im Tourismus für Individualurlaub im Baukastensystem. Das bedeutet auch eine Abkehr von Angeboten und Urlaubsorten, die durch Massentourismus (Overtourism, d. h. zeitliche und räumliche Konzentrationseffekte) gekennzeichnet sind. Immer mehr Menschen wünschen sich im Urlaub ganz spezielle, auf sie persönlich abgestimmte Reiseangebote, die man ggf. auch aus vororganisierten Bausteinen individuell zusammensetzen kann (Zellmann und Mayrhofer 2015; Bandi Tanner und Müller 2019). Exklusivität und Abenteuer werden angestrebt und ein Ort, der noch individuell entdeckt werden kann. Man erwartet maßgeschneiderte Reisen, die Erlebnisse garantieren, durch die der Urlaub einzigartig wird und sich von bisherigen Ferien unterscheidet. Dazu passt auch, dass in der Tourismusbranche das traditionelle Zielgruppenverständnis, insbesondere eine Gruppierung nach demografischen Merkmalen, ausgedient hat. Bereits heute geht es um Erlebnisgruppen, die sich über den jeweiligen Lebensstil definieren. Diese Ausrichtung an der Erlebnisqualität eines Angebots ist unabhängig von Alter, Geschlecht und Einkommen. Die Angebotsbreite wird dadurch schwer überschaubar, doch gleichzeitig kann auf jedes Bedürfnis eingegangen werden (Pröbstl-Haider et al. 2015). In diesem Bereich sind auch Impulse durch neue Technologien zu erwarten.

### Authentische Angebote, attraktive Landschaft, Nachhaltigkeit und Ökolifestyle

Vor dem Hintergrund einer zunehmenden Globalisierung auch der Alltagswelt und spürbarer Auswirkungen des Postfordismus (Wirtschaftsorientierung) in allen gesellschaftlichen Bereichen gewinnen Regionalität und Authentizität als Gegenpole im Tourismus an Bedeutung. Urlaubsdestinationen sollen nicht austauschbar sein. Tourismusveranstalter und Hoteliers müssen ihre ganz speziellen Produkte und die Besonderheiten ihrer Destination daher künftig noch stärker positionieren (Wenzel und Kirig 2006).

Immer wichtiger werden nicht austauschbare Elemente, wie die spezifische Schönheit einer Landschaft, die Sehenswürdigkeiten der Umgebung und lokale Spezialitäten und Traditionen. Von steigender Bedeutung sind in diesem Zusammenhang auch die Sauberkeit der Unterkunft, aber auch die Wasserqualität und die Qualität von Lebensmitteln. Darüber hinaus finden viele Menschen einen Urlaub nur dann entspannend, wenn auch die Sicherheit vor Ort und der Schutz vor Risiken gewährleistet sind. Ein nachhaltiger Lebensstil im Alltag fördert auch den Wunsch nach einem ethischen und umweltfreundlichen touristischen Angebot. Dies gilt allerdings nur, solange andere Aspekte wie Erlebnisorientierung, Genuss, Spaß und Individualität garantiert sind (Bandi Tanner und Müller 2019).

Ein Teilbereich dieser Trends ist auch die sogenannte Respiritualisierung, die Suche nach sinngebenden Elementen im Urlaub, einer starken Natursehnsucht und Erlebnissen, die einen Gegenpol zur Banalität und Funktionalität des Alltags darstellen (Pröbstl-Haider 2015).

Die Entwicklung im Hinblick auf Anpassungsstrategien lässt sich nach Siegrist und Gessner (2011) wie folgt zusammenfassen: Die Mehrzahl der Gäste hat kein Interesse, das eigene Urlaubsverhalten grundsätzlich zu ändern, und will sich nicht „in der schönsten Zeit des Jahres“ mit den Folgen der Klimaerwärmung auseinandersetzen. Anpassungen werden dann akzeptiert, wenn damit nur kleine Einschränkungen verbunden sind, aber nicht die Convenience des Produktes insgesamt betreffen. Die steigende Nachfrage nach authentischen Angeboten im Alpenraum bietet jedoch die Chance für eine klimaschonende Produktenwicklung. Allerdings erwarten Expertinnen und Experten, dass der Schwerpunkt weiterhin auf den bisherigen, konventionellen Angeboten liegen wird. Die Perspektiven für den Skisport werden von einzelnen Autorinnen und Autoren kritisch gesehen, der sich nach Auffassung einzelner Trendforscherinnen und Trendforscher (Zellmann und Mayrhofer 2015) zu einem aufwendigen „Minderheitenprogramm“ entwickeln könnte.

### Alternative Mobilität, Innovation und Digitalisierung

Durch den hohen Anteil touristischer Aktivitäten im ländlichen Raum wird erwartet, dass auch in Zukunft Reisende vor allem für die Vor-Ort-Flexibilität nicht ohne individuelle Mobilitätslösungen auskommen. Auch wenn zu erwarten ist, dass die großen Urlauberströme aus dem Inland und aus Deutschland zunächst noch bevorzugt mit dem eigenen Pkw anreisen, sind alternative Mobilitätskonzepte erforderlich. Diese Entwicklungsperspektiven im Bereich der Mobilität sind im Zusammenhang mit der Informations- und Wissensgesellschaft zu sehen, die nicht nur zu einem anderen

Umgang mit Medien und einer geringeren Bindung an den physischen Raum führt, sondern auch neue Möglichkeiten der Mobilität fördert. Die Digitalisierung im Bereich Verkehr und Buchungsplattformen, die Verknüpfung verschiedener Angebote durch das Smartphone könnten neue Konzepte ermöglichen (Peeters et al. 2016; Sonntag und Lohmann 2019). Neben dem allgemein als bedeutsam eingestuften Einsatz der Elektromobilität diskutiert die Trendforschung vor allem digital vernetzte Modelle (zu den tatsächlichen Entwicklungen im Bereich Verkehr und Transport siehe Kap. 3).

Nachdem die bisherige Dominanz des Pkws in erster Linie aus dem Bedürfnis nach individuellen und Autonomie gewährleistenden Mobilitätslösungen resultiert, müssen neue Lösungen Antworten auf diese Flexibilitätsansprüche finden. Dies erfordert neue Geschäftsmodelle, die eine vernetzte, intermodale Mobilität und attraktive Schnittstellen verschiedener Fortbewegungsarten anbieten. Einzelne Verkehrsmittel stehen dabei nicht länger in Konkurrenz zueinander, sondern ihre Nutzung muss intelligent und innovativ miteinander verzahnt werden. Weitere Optionen entstehen durch das Teilen von Verkehrsmitteln (Sharingangebote). Eine Entwicklung in diesem Bereich wird nicht nur als zu erwartender Trend gesehen, sondern auch als Chance für die Entwicklung von Klimaschutzstrategien (Wippermann 2017). Impulse und Produktentwicklungen entstehen auch durch einen sogenannten digitalen Tourismus, der Innovationen wie digitale Guides oder Apps beinhaltet (Bandi Tanner und Müller 2019).

Die Diskussion mit Tourismusexpertinnen und -experten im Zusammenhang mit den Schließungen aufgrund des Coronavirus im Jahr 2020 ergab, dass eine Reduktion der signifikanten Anstiege der Fern- und Flugreisen erwartet wird. Dazu tragen die erlebten Rückreiseunsicherheiten und die zunehmende Bedeutung von Sicherheits- und gesundheitlichen Aspekten bei. Die dargestellten Trends im Bereich der authentischen Angebote sowie des Gesundheitstourismus könnten sich dadurch verstärken und zu einem vermehrten Urlaub im eigenen Land oder im angrenzenden europäischen Ausland führen.

Vor dem hier dargestellten Ausgangszustand folgen nun im Anschluss Betrachtungen zu den klimatischen Entwicklungen und ihren potenziellen Auswirkungen sowie eine Behandlung aller Reisekomponenten bezogen auf ihren Beitrag zum und ihre Betroffenheit durch den Klimawandel.
